# Increased rhinovirus replication in nasal mucosa cells in allergic subjects is associated with increased ICAM‐1 levels and endosomal acidification and is inhibited by L‐carbocisteine

**DOI:** 10.1002/iid3.102

**Published:** 2016-04-15

**Authors:** Mutsuo Yamaya, Kazuhiro Nomura, Kazuya Arakawa, Hidekazu Nishimura, Nadine Lusamba Kalonji, Hiroshi Kubo, Ryoichi Nagatomi, Tetsuaki Kawase

**Affiliations:** ^1^ Department of Advanced Preventive Medicine for Infectious DiseaseTohoku University Graduate School of MedicineSendai980‐8575Japan; ^2^ Department of Otolaryngology‐Head and Neck SurgeryTohoku University Graduate School of MedicineSendai980‐8575Japan; ^3^ Virus Research Center, Clinical Research DivisionSendai Medical CenterSendai983‐8520Japan; ^4^ Medicine and Science in Sports and ExerciseTohoku University Graduate School of MedicineSendai980‐8575Japan; ^5^ Laboratory of Rehabilitative Auditory ScienceTohoku University Graduate School of Biomedical EngineeringSendai980‐8575Japan

**Keywords:** Airway epithelium, carbocisteine, intercellular adhesion molecule‐1, lung allergy, rhinovirus

## Abstract

Increased viral replication and cytokine production may be associated with the pathogenesis of asthma attacks in rhinovirus (RV) infections. However, the association between increased RV replication and enhanced expression of intercellular adhesion molecule‐1 (ICAM‐1), a receptor for a major RV group, in airway epithelial cells has remained unclear. Furthermore, the inhibitory effects of mucolytics, which have clinical benefits in asthmatic subjects, are uncertain. Human nasal epithelial (HNE) cells were infected with type 14 rhinovirus (RV14), a major RV group. RV14 titers and cytokine concentrations, including interleukin (IL)‐6 and IL‐8, in supernatants, RV14 RNA replication and susceptibility to RV14 infection were higher in HNE cells obtained from subjects in the allergic group (allergic subjects) than in those from subjects in the non‐allergic group (non‐allergic subjects). ICAM‐1 expression and the number and fluorescence intensity of acidic endosomes from which RV14 RNA enters the cytoplasm were higher in HNE cells from allergic subjects, though substantial amounts of interferon (IFN)‐γ and IFN‐λ were not detected in the supernatant. The abundance of p50 and p65 subunits of transcription factor nuclear factor kappa B (NF‐κB) in nuclear extracts of the cells from allergic subjects was higher compared to non‐allergic subjects, and an inhibitor of NF‐κB, caffeic acid phenethyl ester, reduced the fluorescence intensity of acidic endosomes as well as RV titers and RNA. Furthermore, a mucolytic agent, L‐carbocisteine, reduced RV14 titers and RNA levels, cytokine release, ICAM‐1 expression, the fluorescence intensity of acidic endosomes, and NF‐κB activation. The increased RV14 replication observed in HNE cells from allergic subjects might be partly associated with enhanced ICAM‐1 expression and decreased endosomal pH through NF‐κB activation. L‐Carbocisteine inhibits RV14 infection by reducing ICAM‐1 and acidic endosomes and may, therefore, modulate airway inflammation caused by RV infection in allergic subjects.

## Introduction

Rhinoviruses (RVs) are major causes of the common cold that are associated with acute exacerbations of bronchial asthma 1. RV infection induces the production of cytokines, including interleukin (IL)‐1, IL‐6, and IL‐8 2, 3, 4 and mucin and reactive oxygen species from airway epithelial cells 5, 6 and augments airway responsiveness 7. These mechanisms may be related to the development of asthma exacerbation.

Asthmatic patients have bronchial epithelial cells and bronchoalveolar lavage cells that exhibit defective RV‐induced interferon (IFN) production 8. This mechanism has been demonstrated to cause RV infection‐induced asthma exacerbations 9. In contrast, primary cultures of tracheal epithelial cells derived from non‐asthmatic subjects do not produce a significant amount of IFN 4. Therefore, the reason that RV infection causes asthma exacerbation remains unclear because RV infection causes only mild symptoms, such as sore throat and low‐grade fever, in healthy subjects 10. Because intercellular adhesion molecule (ICAM)‐1 is the receptor for the major RV types 11, it has been suggested that the increased ICAM‐1 expression observed in the airway epithelial cells of asthmatic subjects 12, 13 may cause susceptibility to RV infection 13. Based on these findings, we hypothesized that increased levels of ICAM‐1 expression may result in increased RV replication and infection‐induced cytokine production and that these effects may be associated with increased airway inflammation and subsequent asthma exacerbation. However, the relationship between increased levels of ICAM‐1 expression, increased RV replication and increased susceptibility to RV infection in the airway epithelial cells of asthmatic subjects has not been studied. Therefore, in the present study, we compared RV14 replication and RV infection‐induced cytokine production in human nasal epithelial (HNE) cells obtained from allergic subjects, including subjects with bronchial asthma and non‐allergic subjects.

Members of the major RV types enter the cytoplasm of infected cells after binding to their receptor, ICAM‐1 11, 14. The entry of the RNA of a major RV type, RV14, into the cytoplasm of infected cells is thought to be mediated by destabilization that is induced by receptor binding and endosomal acidification 14.

We have previously demonstrated that a mucolytic agent, L‐carbocisteine, inhibits RV infection by reducing ICAM‐1 expression or increasing endosomal pH in human tracheal epithelial cells in subjects who do not have bronchial asthma 15. Furthermore, mucolytic agents have shown clinical benefits in treatments for patients with bronchial asthma, including improvements in tracheobronchial clearance 16, symptoms, and quality of life 17. Mucolytic agents also reduced airway inflammation and hyperresponsiveness in an experimental animal model of bronchial asthma 18. Carbocisteine reduced cough reflexes in asthmatic subjects 19 and reduced mucin production induced by neutrophil elastase and hydrogen peroxide‐induced damage in airway epithelial cells 20, 21. Treatment with carbocisteine also reduced the frequency of asthma exacerbations 22, 23. Based on these findings, it was hypothesized that L‐carbocisteine may also have anti‐inflammatory effects and inhibitory effects on RV replication and RV‐induced cytokine production in the airway epithelial cells of allergic subjects. However, the inhibitory effects of L‐carbocisteine have not been explored.

In the present study, we compared RV14 replication and RV infection‐induced cytokine production in HNE cells obtained from allergic and non‐allergic subjects. We also studied the effect of L‐carbocisteine on RV14 replication and cytokine release in HNE cells infected with RV14.

## Materials and Methods

### Subjects and human nasal epithelial cell culture

The HNE cells used in the culture studies were obtained from nasal specimens excised at the uncinate process during endoscopic endonasal sinus surgeries in the subjects in the allergic group (allergic subjects) (*n* = 15; age, 49 ± 4 years; nine females and six males) and in the subjects in the non‐allergic control group (non‐allergic subjects) (*n* = 15; age, 65 ± 4 years; nine females and six males). The allergic diseases and the reasons for endoscopic surgery in the 30 subjects in the two groups are summarized in Table 1.

**Table 1:** Characteristics of the subjects included in the study

	Allergic group		Non‐allergic group	
Subject number	Allergic diseases and conditions	Reasons for endoscopic surgery	Allergic diseases and conditions	Reasons for endoscopic surgery
1	BA + AS	AS	ND	SC
2	BA + AS	AS	ND	SC
3	BA + AS	AS	ND	SC
4	BA + AS	AS	ND	SC
5	BA + AS + EP	CS	ND	PNC
6	BA + ECS	ECS	ND	PNC
7	BA + ECS	ECS	ND	PNC
8	BA	CS	ND	SM
9	BA	CS	ND	SM
10	BA	CS	ND	SM
11	AS	AS	ND	CS
12	AS	AS	ND	CS
13	AS	AS	ND	CFR
14	ECS + EP	ES	ND	NPC
15	AD	PNC	ND	SBT

AD, atopic dermatitis; AS, allergic sinusitis; BA, bronchial asthma; CS, chronic sinusitis; CFR, cerebrospinal fluid rhinorrhea; ECS, eosinophilic chronic sinusitis; EP, eosinophilia; NPC, nasopharyngeal cancer; PNC, papilloma in nasal cavity; SBT, sphenoid bone tumor; SC, sinus cyst; SM, sinus mycosis; ND, not determined: allergic diseases and conditions were searched, but not found.

**Figure 1:**
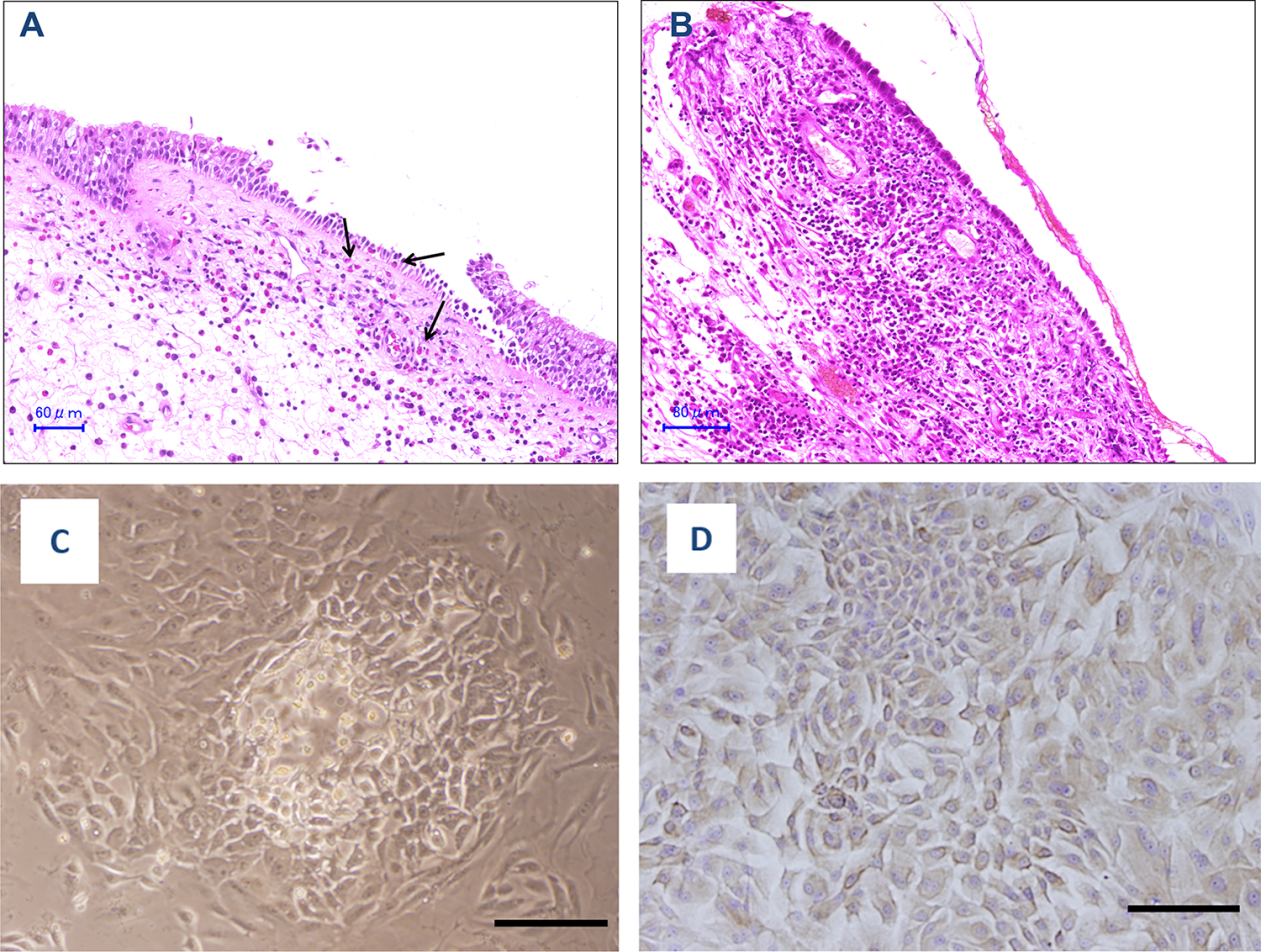
A and B: Hematoxylin‐eosin staining of human nasal mucosal tissues obtained from subjects in the allergic (A) and the non‐allergic (B) groups. The infiltration of eosinophils was observed in the mucosae and submucosae of the tissues obtained from subjects in the allergic group (arrows) (A). C and D: Phase‐contrast microscopy photographs of human nasal epithelial cells at three days (C) and seven days (D) after the initiation of cultures in plastic dishes. Bar = 25 µm.

Of the 15 subjects in the allergic group, 10 had bronchial asthma (Table 1). Of these 10 subjects, five also had allergic sinusitis, and two had eosinophilic chronic sinusitis. Of the five allergic subjects without bronchial asthma, three had allergic sinusitis, one had eosinophilic chronic sinusitis, and one had atopic dermatitis. Two such subjects also had eosinophila greater than 500/μL in the peripheral venous blood (Table 1). The specific serum IgE antibody levels were greater than 170 IU/mL for at least one common inhalant allergen in all of the subjects in the allergic group. Furthermore, significant infiltration by eosinophils was observed in the mucosa and submucosa in tissues obtained from allergic subjects, as shown in Figure 1A. Bronchial asthma was defined according to the 2015 Global Initiative for Asthma (GINA) guidelines 24.

In contrast, none of the 15 subjects in the non‐allergic control group had any allergic disease (Table 1), such as allergic nasal or sinus diseases. None of the subjects in the non‐allergic group had high serum IgE antibody levels or eosinophilia. Furthermore, no significant infiltration of eosinophils was observed in the mucosa and submucosa in the tissues obtained from non‐allergic subjects, as shown in Figure 1B. The reasons for endoscopic nasal surgery in the subjects in the non‐allergic group were a sinus cyst (*n* = 4), papilloma in the nasal cavity (*n* = 3), sinus mycosis (*n* = 3), chronic sinusitis (*n* = 2), cerebrospinal fluid rhinorrhea (*n* = 1), nasopharyngeal cancer (*n* = 1), and a sphenoid bone tumor (*n* = 1) (Table 1).

None of the patients was being treated with L‐carbocisteine at the time of surgery. This study was approved by the Tohoku University Ethics Committee.

For the HNE cell culture experiments, surface epithelial cells were isolated from human nasal mucosae using 0.05% protease and then cultured in plastic tubes (Becton Dickinson, Franklin Lakes, NJ), 24‐well plates, on coverslips in Petri dishes, or on glass slides as previously described 25, 26, 27.

### Human embryonic fibroblast cell culture

Human embryonic fibroblast cells (HEF, HFL‐III cells; Riken Bio Resource Center Cell Bank, Cell No: RCB0523; Tsukuba, Japan) were cultured as previously described 26.

### Immunocytochemistry of nasal epithelial cells

To confirm the presence of epithelial cells, the isolated cells that presumably contained nasal epithelial cells were cultured on slide glasses, fixed with ethanol and stained using an anti‐keratin antibody (MAK‐5; Triton, Alameda, CA) as previously described 27. The cells were then incubated with biotinylated horse anti‐mouse immunoglobulins (Vector Laboratories, Burlingame, CA) followed by incubation with avidin–biotin peroxidase complexes (Vectastain ABC Kit; Vector Laboratories). Immunocytochemistry was also performed using cultured cells that were isolated from nasal specimens or human embryonic fibroblasts (HEFs) using an anti‐vimentin antibody (DAKO, Santa Barbara, CA) to exclude fibroblast contamination or to confirm the presence of fibroblasts, respectively 27.

### Viral stock, viral titration, and susceptibility to rhinovirus infection

An RV14 stock was prepared from a patient with the common cold by infecting HEF cells as previously described 28, with some modifications (for e.g., we used plastic tubes instead of microplates). RV14 was identified using a micro‐neutralization test with an antibody for RV14 as previously described 29. RV14 stocks were generated by infecting HEF cells. The cells were then cultured in plastic tubes in 1 mL of MEM supplemented with 2% ultra‐low IgG calf serum and antibiotics at 33°C. The cells were incubated for several days until cytopathic effects were obvious, after which the cultures were frozen at −80°C, thawed, and sonicated. The virus‐containing fluid obtained from these cells was frozen in aliquots at −80°C. We used RV14 stocks that had been passaged 3–5 times.

The RV14 that was present in the supernatants (the cell‐culture medium) was titrated using HEF cells with endpoint methods 26, 30. The rate of RV14 release into the supernatant was expressed as TCID_50_ (tissue culture infective dose) units/mL/24 h for each sample 26. Cellular susceptibility to RV14 infection was evaluated as previously described 26.

### Viral infection of epithelial cells

HNE cells were infected with RV14 (100 μL in each tube, 1.0 × 10^4^ TCID_50_ units/100 µL, 5.0 × 10^−2^ TCID_50_ units/cell) using previously described methods 31.

### Quantification of rhinoviral RNA

To quantify the amount of RV14 RNA and the β‐actin mRNA expression levels in the HNE cells, a two‐step real‐time quantitative reverse transcription (RT)‐PCR assay was performed using TaqMan^®^ Gene Expression Master Mix (Applied Biosystems, Bedford, CA) as described by Nolan et al. 32 and according to previously described methods 31, with some modification. The housekeeping gene β‐actin was used as the control.

The RNA extracted from the cells was divided into 10 ng aliquots, and the amount of RV14 RNA was measured in each 10 ng aliquot of RNA that was extracted from the same cells. To measure the amount of RV14 RNA and β‐actin mRNA in the samples, the RNA that was extracted from the cells was converted to cDNA, and the amount of cDNA for the RV14 RNA and β‐actin mRNA was then measured. TaqMan technology exploits the 5′‐3′ nucleolytic activity of AmpliTaq Gold^®^ DNA Polymerase. In principle, this method uses a dual‐labeled fluorogenic hybridization probe, the TaqMan probe, which specifically anneals the template between the PCR primers.

In the first step during the quantification of RV14 RNA, the cDNA from RV14 RNA was constructed using a QuantiTect Reverse Transcription Kit (Qiagen, Germanton, MD). Briefly, an extracted aliquot containing RNA (10 ng/5 µL) was mixed with gDNA Wipeout Buffer (2 µL) and RNAase‐free water (2 µL) and incubated at 42°C for 2 min. The mixture (9 µL) was then mixed with Quantiscript Reverse Transcriptase (1 µL), Quantiscript RT Buffer (4 µL), RT Primer Mix (1 µL), and an RV reverse primer (5′‐CGGACACCCAAAGTAGTCGGT‐3′; 5 µL) to obtain a cDNA for the RV14 RNA. This mixture was incubated at 42°C for 15 min and then at 95°C for 3 min using a GeneAmp^®^ PCR System 9700 (Applied Biosystems).

Similarly, in the second step during the quantification of RV14 RNA, real‐time PCR was performed using cDNA constructed from RV14 RNA using TaqMan® Gene Expression Master Mix. The probe contained a fluorescent reporter (6‐carboxyfluorescein [FAM]) at its 5′ end and a fluorescent quencher (6‐carboxytetramethylrhodamine [TAMRA]) at its 3′ end. As the PCR progresses, the TaqMan probe is degraded to release the reporter, resulting in an increase in fluorescence emission. We used a sequence detector (ABI PRISM 7700; Applied Biosystems) to measure the amount of amplified product, which was indicated in direct proportion by the continuous increase in fluorescence emission during PCR. Briefly, the cDNA sample (2 µL) made in the first step was mixed with TaqMan Gene Expression Master Mix (10 μL), forward primer (5′‐GCACTTCTGTTTCCCAGGAGC‐3′; 0.5 µL), reverse primer (5′‐CGGACACCCAAAGTAGTCGGT‐3′; 0.5 µL), a TaqMan RV14 probe (5′‐[FAM] CCTTTAACCGTTATCCGCCA [TAMRA]‐3′; 0.5 µL), and RNAase‐free water (6.5 µL). The cDNA made from RV14 RNA was amplified using PCR for 45 cycles (15 sec at 95°C and 1 min at 60°C). We used the PrimerExpress program (version 3.0; Applied Biosystems) to design the probe and primers according to the guidelines for obtaining the best performance in the PCR. The TaqMan RV14 probe was designed for RV14 based on a previous report 4. Whole RT‐PCRs and detection of the fluorescence emission signal during each PCR cycle were performed at the same time using a single tube in a sequence detector (ABI 7700).

The RT‐PCR for β‐actin was also performed as previously described 33. The expression of RV14 RNA was normalized to the constitutive expression of β‐actin mRNA.

### Measurement of ICAM‐1 expression

The level of ICAM‐1 mRNA was examined using two‐step real‐time RT‐PCR analysis according to the methods described above (Quantification of rhinoviral RNA) with designed forward (5′‐GCACTTCTGTTTCCCAGGAGC‐3′) and reverse (5′‐CGGACACCCAAAGTAG TCGGT‐3′) primers 31. A TaqMan probe (5′‐[FAM] CCTTTAACCGTTATCCGCCA [TAMRA]‐3′] was designed for ICAM‐1. The expression of ICAM‐1 mRNA was also normalized to the constitutive expression of β‐actin mRNA. The concentration of the soluble form of ICAM‐1 (sICAM‐1) in the supernatants was measured using an enzyme‐linked immunosorbent assay (ELISA) (BioLegend, San Diego, CA).

### Measurement of changes in acidic endosomes

The distribution and fluorescence intensity of acidic endosomes in the cells were measured as previously described using LysoSensor DND‐189 dye (Molecular Probes, Eugene, OR) 31.

Live‐cell imaging was performed. The cells were cultured on coverslips in Petri dishes and observed using a fluorescence microscope (OLYMPUS IX70; OLYMPUS Co. Ltd., Tokyo, Japan). The excitation wavelength was 443 nm, and the emitted light from the cells was detected using a 505‐nm filter. Fluorescence intensities were calculated using a fluorescence image analyzer system (Lumina Vision®; Mitani Co. Ltd., Fukui, Japan) that was equipped with a fluorescence microscope. The fluorescence intensity of acidic endosomes was measured in 100 HNE cells, and the mean value of the fluorescence intensities was expressed as the percentage of the control value and compared to the fluorescence intensity of the cells that were pretreated with vehicle, as described below.

### Measurement of cytokine production

The levels of IL‐1β, IL‐6, IL‐8, IFN‐γ, and IFN‐λ in the supernatants were measured using ELISAs, and tumor necrosis factor (TNF)‐α levels were measured using a chemiluminescent enzyme immunoassay.

### Assay of the nuclear factor kappa‐B

The presence of the p50 and p65 subunits of nuclear factor kappa‐B (NF‐κB) in the nuclear extracts was assayed using a TransAM™ NF‐κB Family (Active Motif, Carlsbad, CA) according to the manufacturer's instructions and as previously described 26.

### Pretreatment with L‐carbocisteine

To examine the effects of L‐carbocisteine, HNE cells were pretreated with either L‐carbocisteine (10 μM unless otherwise described; Kyorin Pharmaceutical Co. Ltd., Tokyo, Japan) or vehicle (0.1% double‐distilled water) as previously described 15. According to the protocols provided by Kyorin Pharmaceutical Co. Ltd., L‐carbocisteine was dissolved in 1N NaOH to obtain a solution with a concentration of 10^−1^ M. The solution of L‐carbocisteine was neutralized by adding 1N HCl to obtain a pH of 7.0 and then diluted to a concentration of 10^−2^ M by adding double‐distilled water. To make a culture medium containing 10^−5^ M of L‐carbocisteine, 10 µL of 10^−2^ M L‐carbocisteine solution was diluted 1000‐fold by mixing it with 10 mL of culture medium. Because the solution of L‐carbocisteine was neutralized by adding HCl and then diluted with water, 10 µL of double‐distilled water was mixed with 10 mL of culture medium to make the culture medium that contained the vehicle for L‐carbocisteine.

In preliminary experiments, we found that the maximum inhibitory effect of L‐carbocisteine on RV14 titers was observed when the HNE cells were pre‐treated with L‐carbocisteine for three days (72 h) or longer prior to infection (data not shown). Therefore, in the present study, the cells were pretreated with L‐carbocisteine for three days prior to infection with RV14 and then treated until the end of the experimental period (after RV14 infection), as reported in a previous study that used human tracheal epithelial cells 15.

Furthermore, the HNE cells were pretreated with L‐carbocisteine for 72 h to examine the effects of L‐carbocisteine on ICAM‐1 mRNA expression, the concentration of sICAM‐1, and the pH of the endosomes in the absence of RV14 infection.

To examine the concentration‐dependent effects of L‐carbocisteine on RV14 infection, the HNE cells were pretreated with L‐carbocisteine at concentrations ranging from 0.01 to 100 µM.

To confirm the mechanism by which viral infection was inhibited by L‐carbocisteine, the HNE cells were pretreated with an inhibitor of NF‐κB, caffeic acid phenethyl ester (CAPE; Calbiochem, La Jolla, CA; 10 µM, 72 h) 34, the specific vacuolar H^+^‐ATPase inhibitor bafilomycin A_1_ (0.1 µM, 72 h) 35, 36, or vehicle (0.05% dimethylsulfoxide; DMSO). The HNE cells used in this set of experiments were obtained from non‐allergic subjects (*n* = 3, age, 54 ± 14 years, three males with nasopharyngeal cancer) who were different from the 30 subjects shown in Table 1.

### Collection of supernatants for measurements

We collected the supernatants at day 0, prior to RV14 infection, to evaluate cytokine release and the concentration of sICAM‐1. Furthermore, to examine the viral release and secretion of cytokines, including IL‐6 and IL‐8, the supernatants of the HNE cells obtained from allergic and non‐allergic subjects were collected at one (24 h), three (72 h), and five days (120 h) after infection using previously described methods 15. We also examined the effects of L‐carbocisteine on viral release and cytokine secretion using the supernatants collected at the same post‐infection time‐points.

### Statistical analysis

The results are expressed as the mean ± SEM. To compare data between the two groups, Student's *t*‐tests or Mann‐Whitney *U*‐tests were performed. To compare data between more than two groups, the statistical analyses were performed using analysis of variance (ANOVA), and subsequent post hoc analyses were performed using Bonferroni's method. For all analyses, values of *P* < 0.05 were considered to be significant. The number of subjects from whom the nasal specimens were obtained is referred to as *n*.

## Results

### Histological characteristics of human nasal epithelium

Hematoxylin‐eosin staining of nasal tissues obtained from subjects in the allergic group (allergic subjects) showed that the mucosae and submucosae of these subjects were infiltrated with eosinophils (Fig. 1A). In contrast, lymphocytes, histiocytes, and neutrophils were observed in the mucosae and submucosae in the nasal tissues obtained from subjects in the non‐allergic group (non‐allergic subjects) (Fig. 1B).

### Growth and immunocytochemical examination of human nasal epithelial cells

Cells and cell clusters, which were isolated using a protease treatment, were adhered to culture vessels in plastic tubes, plastic dishes (Fig. 1C), on coverslips in Petri dishes, or glass slides and the cells then began to proliferate. Contaminating blood cells, fibers and debris did not attach to the culture vessels and were removed by changing the medium several times. Next, the HNE cells began to spread and they became confluent 7–10 days after plating (Fig. 1D).

**Figure 2:**
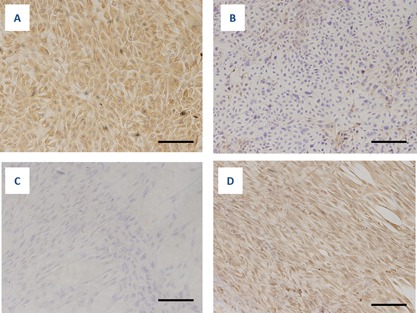
Immunocytochemistry of cultured cells isolated from nasal mucosal tissues (A and B) and human embryonic fibroblast (HEF) cells (C and D). Experiments were performed using monoclonal antibodies directed against epithelial cells (anti‐keratin antibody; A and C) and fibroblasts (anti‐vimentin antibody; B and D). Nasal mucosa cells (=HNE cells) were positively stained by an anti‐keratin antibody but not an anti‐vimentin antibody (A and B), although weak non‐specific staining was observed for the anti‐vimentin antibody (B). Bar = 25 µm.

Immunocytochemical examination of the cultured HNE cells showed that the cells were positively labeled by an anti‐keratin antibody (Fig. 2A) but no staining was observed when an anti‐vimentin antibody was applied (Fig. 2B). In contrast, human embryonic fibroblast (HEF) cells did not stain when an anti‐keratin antibody was used but were positively labeled by the anti‐vimentin antibody (Fig. 2C and D).

### Rhinovirus replication and susceptibility to infection in nasal epithelial cells obtained from allergic subjects

**Figure 3:**
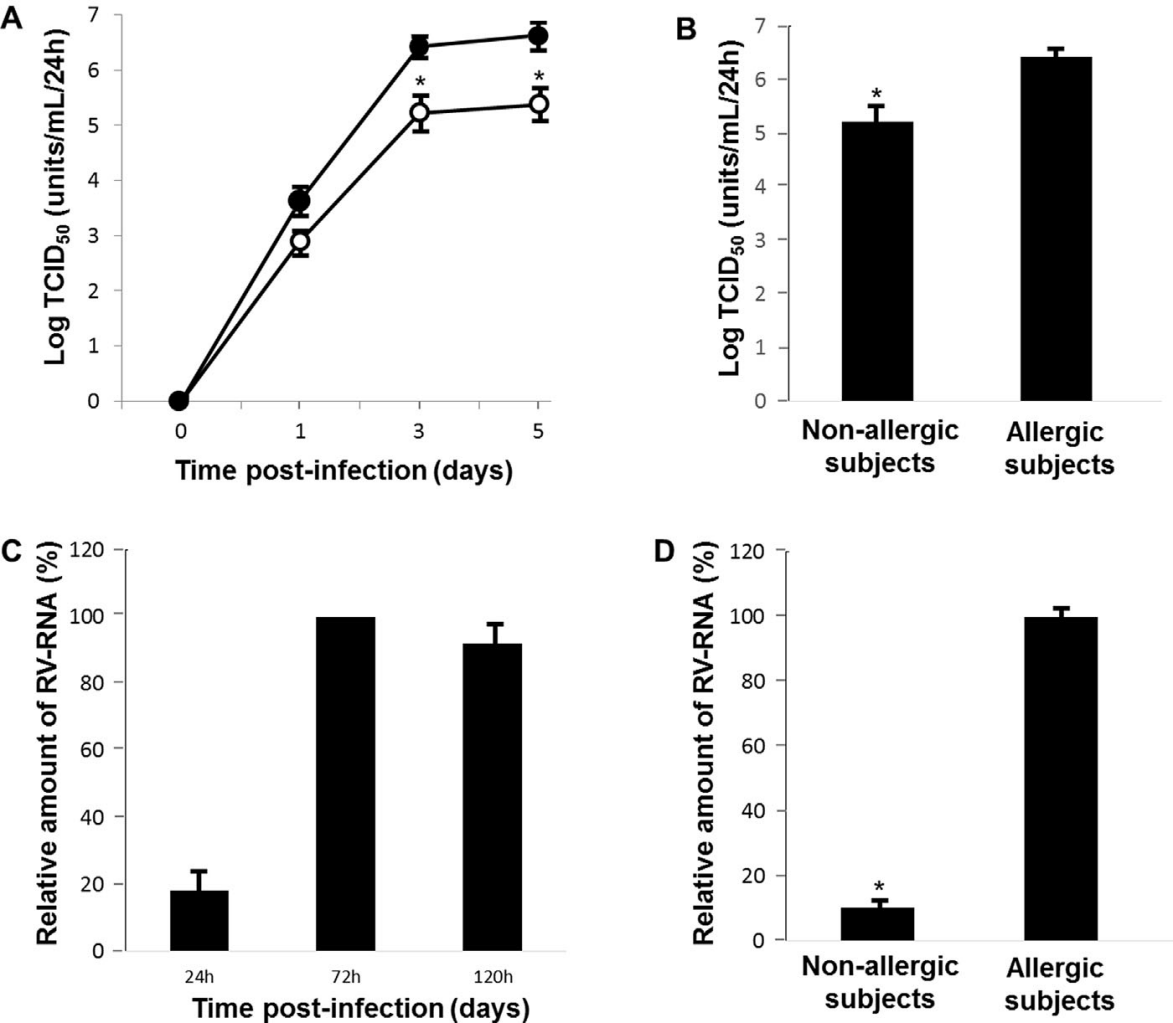
A: The time courses of viral release into supernatants by HNE cells obtained from allergic (closed circles) and non‐allergic (open circles) subjects. Different time points after RV14 infection are shown, as indicated. B: Viral release into supernatants collected at 72 h after infection. The cultured cells were obtained from subjects in the allergic (allergic subjects) or non‐allergic (non‐allergic subjects) group. C: Time courses showing RV14 RNA replication in the cells obtained at different times after RV14 infection from allergic subjects. D: Replication of RV14 RNA at 72 h after infection in the cells obtained from allergic or non‐allergic subjects. A–D: The results are presented as the mean ± SEM for nine (A, B, and D) or three (C) subjects. The results showing RV14 RNA levels (C and D) are expressed as the relative amount of RNA expression (%) compared to the mean value of the peak level of RV14 RNA expression at 72 h after infection in the cells obtained from allergic subjects. Significant differences from the cells obtained from allergic subjects are indicated by *P < 0.05.

Exposing confluent HNE cells to RV14 consistently led to infection. No virus was detected at 1 h after infection, but RV14 was detected in the supernatants at 24 h (Fig. 3A). Evidence of continuous viral replication was obtained in experiments that demonstrated that each of the supernatant samples collected at either 24, 72, or 120 h after infection contained significant levels of RV14 (Fig. 3A). The viral titer levels in the supernatants increased significantly with time for the first 72 h after infection.

The viral titers in the supernatants at 72 and 120 h after infection of the HNE cells of allergic subjects were significantly higher than the titers in the cells obtained from non‐allergic subjects (Fig. 3A and B). The viral titers in the supernatants of the HNE cells of allergic non‐asthmatic subjects were also significantly higher than the titers in the cells obtained from non‐allergic subjects (6.6 ± 0.2 vs. 5.4 ± 0.2 TCID_50_ units/mL/24 h, *n* = 5, *P *< 0.05). No virus was detected in any of the supernatants after infection with ultraviolet (UV)‐inactivated RV14 (data not shown).

RV14 RNA was consistently observed in the HNE cells from 24 h after infection, and these RNA levels increased between 24 and 72 h after infection (Fig. 3C). The peak level of RV14 RNA replication was observed at 72 h after infection (Fig. 3C), whereas RV14 RNA was not observed before infection.

At 72 h after infection, the RV14 RNA levels in the HNE cells obtained from allergic subjects were higher than the levels in the HNE cells obtained from non‐allergic subjects (Fig. 3D). The RV14 RNA levels in the HNE cells obtained from allergic non‐asthmatic subjects were also higher than the levels in the cells obtained from non‐allergic subjects (100.0 ± 2.4 vs. 11.3 ± 1.3%, *n* = 5, *P *< 0.05; the relative amount of RNA expression [%] compared with the mean value of the peak level of RV14 RNA expression in the cells obtained from allergic non‐asthmatic subjects).

Susceptibility to RV14 infection was increased in the HNE cells obtained from allergic subjects. The minimum dose of RV14 required to cause infection was significantly lower in the cells obtained from allergic subjects than the dose required to infect the cells obtained from non‐allergic subjects (2.6 ± 0.1 vs. 3.5 ± 0.2 TCID_50_ units/mL, *n* = 5, *P* < 0.05).

### ICAM‐1 and acidic endosomes in nasal epithelial cells obtained from allergic subjects

**Figure 4:**
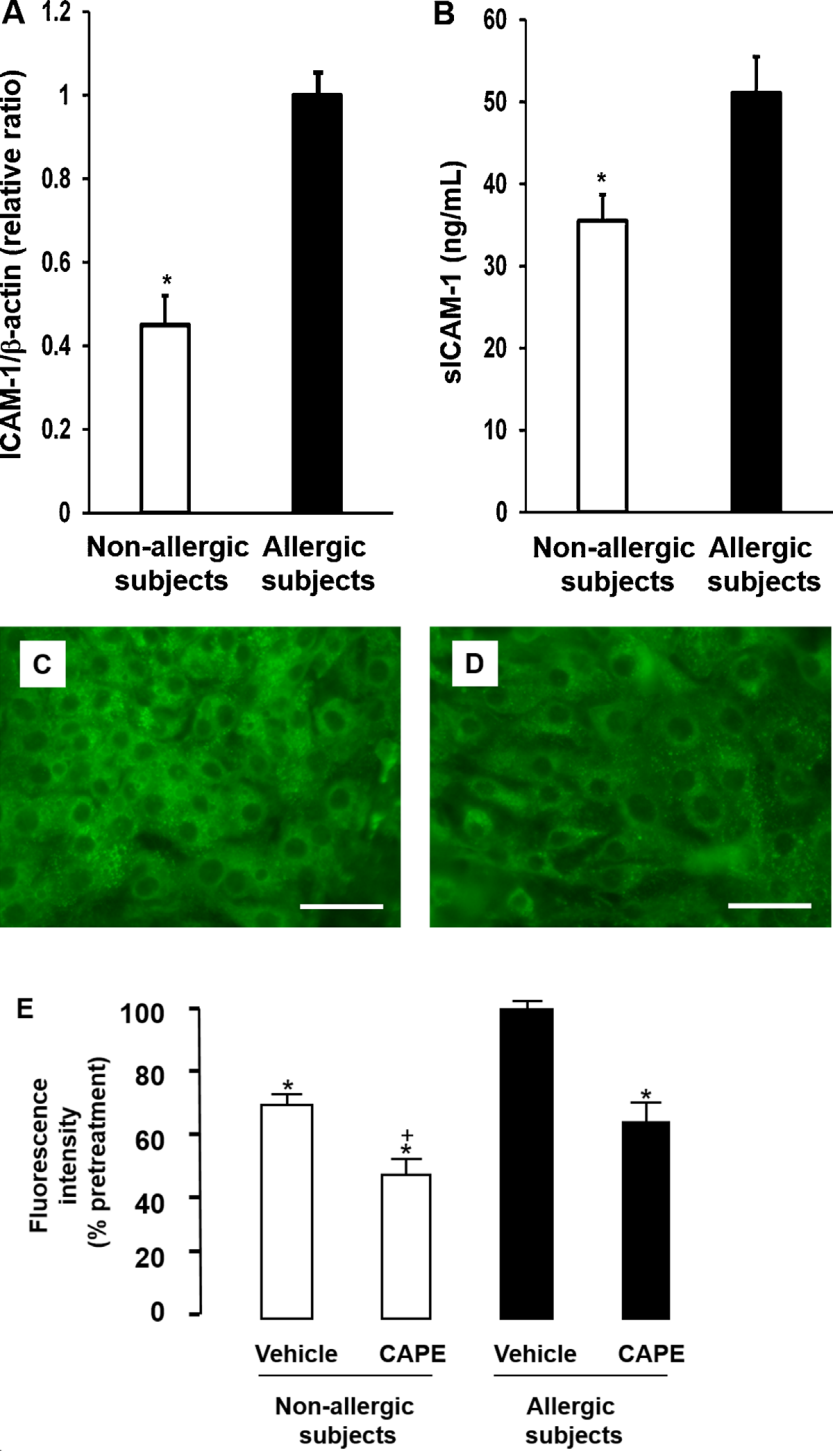
A and B: Expression of ICAM‐1 mRNA (A) and sICAM‐1 concentrations in the supernatants (B) before RV14 infection in the HNE cells from subjects in the allergic (allergic subjects) or non‐allergic (non‐allergic subjects) group. The expression of ICAM‐1 mRNA was normalized to the constitutive expression level of β‐actin mRNA. The mean value for ICAM‐1 mRNA expression levels in the cells obtained from allergic subjects was set to 1.0. The results are presented as the mean ± SEM from five subjects. Significant differences from the values obtained for the cells in the allergic subjects are indicated by *P < 0.05. C and D: Distribution of acidic endosomes exhibiting green fluorescence in HNE cells obtained from allergic (C) or non‐allergic (D) subjects. Data are representative of four different experiments (Bar = 100 µm). E: The fluorescence intensity of acidic endosomes in HNE cells obtained from allergic (black bars) or non‐allergic (white bars) subjects at 72 h after pretreatment with CAPE (10 µM) or vehicle (0.05% DMSO). The mean value of fluorescence intensity observed in the vehicle‐pretreated cells obtained from allergic subjects was set to 100%. The results are presented as the mean ± SEM from five subjects. Significant differences from the values obtained for the vehicle‐pretreated cells obtained from allergic or non‐allergic subjects are indicated by *P < 0.05 and ^+^ *P* < 0.05, respectively.

The baseline ICAM‐1 mRNA expression in HNE cells obtained from allergic subjects was significantly higher than the baseline levels in the cells obtained from non‐allergic subjects prior to infection (Fig. 4A). Similarly, the sICAM‐1 concentrations in the supernatants of HNE cells obtained from allergic subjects were significantly higher than those in the supernatants of cells obtained from non‐allergic subjects (Fig. 4B).

The number (Fig. 4C and D) and fluorescence intensity (Fig. 4C–E) of acidic endosomes were significantly higher in the HNE cells obtained from allergic subjects than in the cells obtained from non‐allergic subjects. Furthermore, an inhibitor of NF‐κB, CAPE (10 µM, 72 h) reduced the fluorescence intensity of acidic endosomes (Fig. 4E).

### Cytokine production in nasal epithelial cells obtained from allergic subjects

Prior to infection, the baseline levels of IL‐6 and IL‐8 secretion were significantly higher in the HNE cells obtained from allergic subjects than in the cells obtained from non‐allergic subjects (Table 2). RV14 infection increased the secretion of IL‐6 and IL‐8 into the supernatants (Table 2). The maximum level of secretion into the supernatants was observed at 72 h after infection for both IL‐6 and IL‐8 (data not shown). Furthermore, after infection, the maximum level of secretion of these cytokines by the HNE cells obtained from allergic subjects was significantly higher than that in the cells obtained from non‐allergic subjects (Table 2). We detected only small amounts of IL‐1β (less than 50 pg/mL) and TNF‐α (less than 100 pg/mL), and the level of TNF‐α secretion was not different between the allergic and non‐allergic subjects. Significant amounts of IFN‐γ and IFN‐λ were not detected in the supernatants.

**Table 2:** Cytokine release in human nasal epithelial obtained cells from allergic or non‐allergic subjects before and after RV infection and the effects of L‐carbocisteine

	Cells from non‐allergic subjects		Cells from allergic subjects	
	Before RV infection	After RV infection	Before RV infection	After RV infection
IL‐6 (ng/mL)				
Vehicle	1.7 ± 0.2	5.9 ± 0.7*	4.8 ± 0.3*	32.9 ± 2.5^**++‡^
L‐CC	1.2 ± 0.1*	3.3 ± 0.3^*‡^	3.8 ± 0.3^*+^	21.2 ± 2.1^**++‡§^
IL‐8 (ng/mL)				
Vehicle	20.6 ± 1.7	51.3 ± 4.9*	40.8 ± 3.2*	98.3 ± 7.6^**+‡^
L‐CC	16.3 ± 0.7*	25.0 ± 1.8^‡^	32.4 ± 2.4^*+^	63.4 ± 5.3^**+§^

The levels of cytokines in the supernatants of human nasal epithelial cells obtained from subjects in the allergic or non‐allergic group before and at 72 h after RV14 infection. Cells were pretreated with L‐carbocisteine (L‐CC; 10 µM) or vehicle alone for 72 h before infection or treated with L‐CC or vehicle alone beginning three days before infection and continuing until 72 h after infection. The results are presented as the mean ± SEM of five samples. Significant differences compared to cells obtained from the non‐allergic subjects that were pretreated with the vehicle alone (Vehicle) prior to infection are indicated by *P < 0.05 and **P < 0.01. Significant differences compared to cells obtained from allergic subjects pretreated with the vehicle alone (Vehicle) prior to infection are indicated by ^+^ *P* < 0.05 and ^++^ *P* < 0.01. Significant differences compared to vehicle‐pretreated cells obtained from non‐allergic subjects at 72 h after RV14 infection are indicated by ^‡^ *P* < 0.05. Significant differences compared to vehicle‐pretreated cells obtained from allergic subjects at 72 h after RV14 infection are indicated by ^§^ *P* < 0.05.

### NF‐kappa B in nasal epithelial cells obtained from allergic subjects

Prior to infection, the amount of p50 and p65 of NF‐κB in nuclear extracts was greater in the HNE cells obtained from allergic subjects than in the cells obtained from non‐allergic subjects (Table 3). The amount of p50 and p65 of NF‐κB increased after RV14 infection, and the amount of these proteins in the cells obtained from allergic subjects was also greater than the amount in the cells obtained from non‐allergic subjects after infection (Table 3).

**Table 3:** NF‐κB activation in human nasal epithelial cells obtained from allergic or non‐allergic subjects before and after RV infection and the effects of L‐carbocisteine

	Cells from non‐allergic subjects		Cells from allergic subjects	
	Before RV infection	After RV infection	Before RV infection	After RV infection
p50				
Vehicle	0.30 ± 0.01	0.76 ± 0.04*	0.39 ± 0.02*	1.08 ± 0.07^**++‡^
L‐CC	0.16 ± 0.01*	0.59 ± 0.03^*‡^	0.19 ± 0.02^+^	0.69 ± 0.04^**+§^
p65				
Vehicle	0.20 ± 0.01	0.38 ± 0.02*	0.25 ± 0.01*	0.61 ± 0.04^*+‡^
L‐CC	0.11 ± 0.01*	0.30 ± 0.01^*‡^	0.13 ± 0.01^+^	0.48 ± 0.03^*+§^

Levels of p50 and p65 in the nuclear extracts of HNE cells obtained from subjects in the allergic or non‐allergic group. Levels are shown before infection and at 72 h after RV14 infection. Cells were pretreated with L‐carbocisteine (L‐CC; 10 µM) or vehicle alone for 72 h before infection or pretreated with L‐CC or vehicle alone beginning three days before infection and continuing until 72 h after infection. The results are expressed as the optical density (OD) and the mean ± SEM of five samples. Significant differences compared to the cells obtained from non‐allergic subjects that were pretreated with the vehicle alone (Vehicle) prior to infection are indicated by *P < 0.05 and **P < 0.01. Significant differences compared to the cells obtained from allergic subjects that were pretreated with the vehicle alone (Vehicle) prior to infection are indicated by ^+^ *P* < 0.05 and ^++^ *P* < 0.01. Significant differences compared to the vehicle‐pretreated cells obtained from non‐allergic subjects at 72 h after RV14 infection are indicated by ^‡^ *P* < 0.05. Significant differences compared to vehicle‐pretreated cells obtained from allergic subjects at 72 h after RV14 infection are indicated by ^§^ *P* < 0.05.

### Effects of L‐carbocisteine on rhinovirus replication

**Figure 5:**
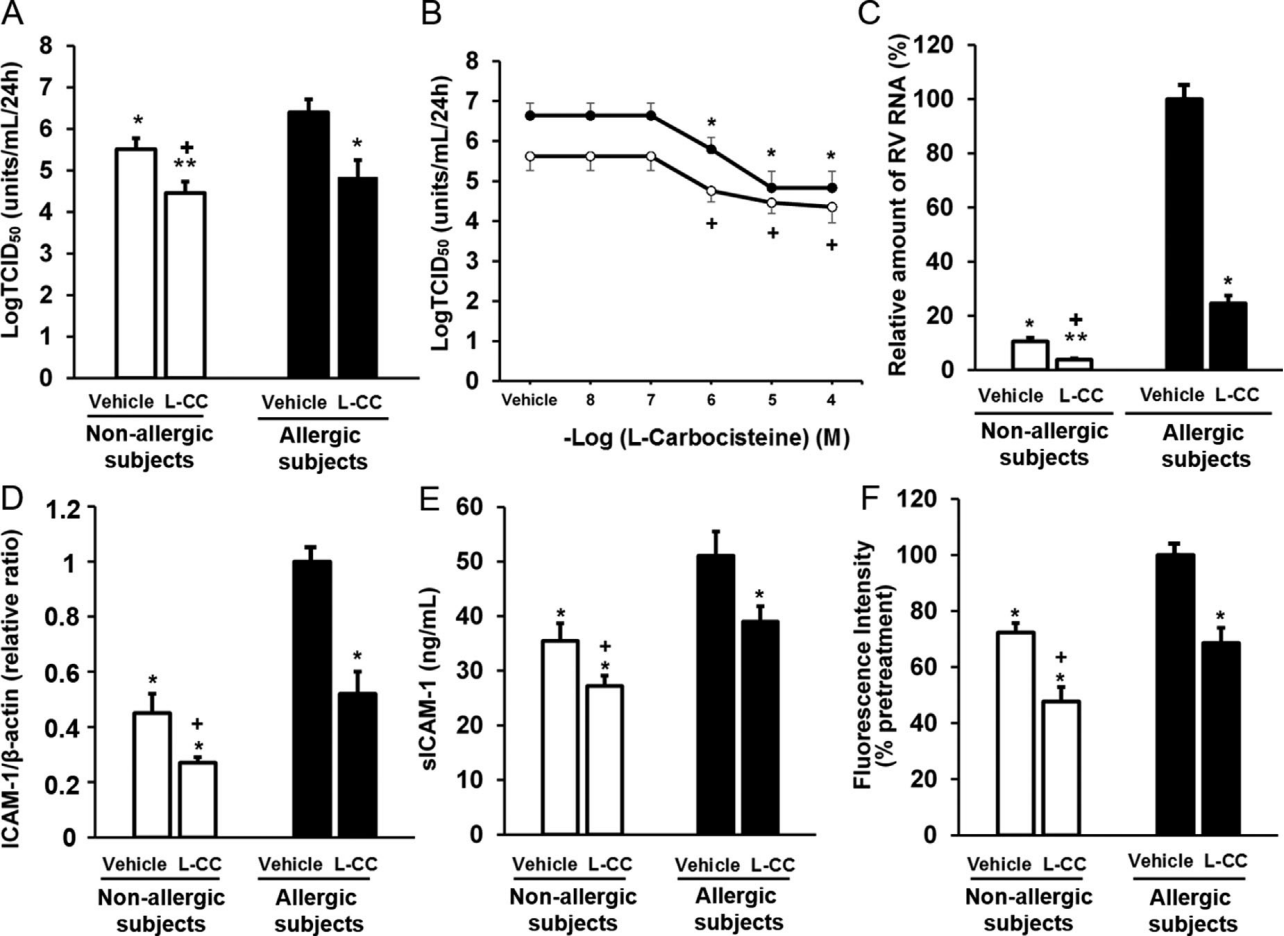
A: Viral release into the supernatants collected at 72 h after infection in the presence of L‐carbocisteine (10 µM, L‐CC) or vehicle of L‐carbocisteine (Vehicle, 0.1% of water) in the HNE cells obtained from subjects in the allergic or non‐allergic group (allergic or non‐allergic subjects). B: The concentration‐dependent effect of L‐carbocisteine on the viral release into the supernatants collected at 72 h after infection in the HNE cells obtained from allergic (closed circles) or non‐allergic (open circles) subjects. C: The replication of RV14 RNA at 72 h after infection in the presence of L‐carbocisteine (L‐CC) or vehicle in the HNE cells obtained from allergic or non‐allergic subjects. The results are expressed as the relative amount of RNA (%) compared to the mean value of the peak level of RV14 RNA in the vehicle‐pretreated cells obtained from allergic subjects. D and E: The expression of ICAM‐1 mRNA in the HNE cells (D) and sICAM‐1 concentrations in the supernatants (E) prior to RV14 infection in the presence of pretreatment with L‐carbocisteine (L‐CC) or vehicle in the cells obtained from allergic or non‐allergic subjects. The mean value for ICAM‐1 mRNA expression levels in the vehicle‐pretreated cells obtained from allergic subjects was set to 1.0. F: The fluorescence intensity of acidic endosomes in the presence of pretreatment with L‐carbocisteine (L‐CC) or vehicle for 72 h in the HNE cells obtained from allergic or non‐allergic subjects. The mean value for the fluorescence intensity in the vehicle‐pretreated cells obtained from allergic subjects was set to 100%. A–F: The results are presented as the mean ± SEM from nine (A and B), eight (C), or five (D–F) subjects. Significant differences from the vehicle‐pretreated cells obtained from allergic subjects are indicated by *P < 0.05 and **P < 0.01. Significant differences from the vehicle‐pretreated cells obtained from non‐allergic subjects are indicated by ^+^ *P* < 0.05.

Pretreating the cells with L‐carbocisteine (10 µM) significantly decreased the viral titers of RV14 in supernatants at 72 h after infection in the cells obtained from subjects in both the allergic and non‐allergic groups compared to the titers observed in the cells pretreated with vehicle (Fig. 5A). Treatment with L‐carbocisteine at concentrations of 1 µM or greater reduced RV14 release in a concentration‐dependent manner in the cells from both groups of subjects (Fig. 5B). L‐carbocisteine also decreased RV14 RNA levels at 72 h after infection in the cells obtained from both groups of subjects (Fig. 5C).

CAPE and bafilomycin A_1_ reduced RV14 titers in the culture supernatants of cells obtained from non‐allergic subjects (4.5 ± 0.1 for CAPE and 2.4 ± 0.2 for bafilomycin A_1_ vs. 5.5 ± 0.2 TCID_50_ units/mL/24 h for the vehicle [0.05% DMSO]; *n* = 3, *P* < 0.05). CAPE and bafilomycin A_1_ also reduced RV14 RNA levels (21.3 ± 1.1% for CAPE and 2.4 ± 0.2% for bafilomycin A_1_, shown as the relative amount of RNA [%] compared to the mean value of the peak level of RV14 RNA in the vehicle‐pretreated cells at 72 h after infection, *n* = 3, *P* < 0.05).

Cell viability was assessed by analyzing trypan blue exclusion assays and lactate dehydrogenase (LDH) concentrations in the supernatants of cells at 72 h after L‐carbocisteine treatment. No difference in cell viability was observed (data not shown).

We also studied the effects of L‐carbocisteine on ICAM‐1 expression and acidic endosomes to explore the mechanisms that might underlie the inhibitory effects of L‐carbocisteine on RV14 replication. Prior to infection, ICAM‐1 mRNA expression and the concentrations of sICAM‐1 were significantly lower in the cells that were pretreated with L‐carbocisteine than in the cells that were pretreated with vehicle in both groups of subjects (Fig. 5D and E). Pretreatment with L‐carbocisteine also reduced the fluorescence intensity of the acidic endosomes in the cells (Fig. 5F).

### Effects of L‐carbocisteine on cytokine production

In the HNE cells obtained from both groups of subjects, pretreatment with L‐carbocisteine (72 h) reduced the baseline secretion of IL‐6 and IL‐8 prior to RV14 infection (Table 2). RV14 infection increased the secretion of IL‐6 and IL‐8, and pretreatment with L‐carbocisteine reduced the RV14 infection‐induced secretion of these cytokines in the cells obtained from both groups of subjects (Table 2).

### Effects of L‐carbocisteine on NF‐kappa B

In the HNE cells obtained from both groups of subjects, pretreatment with L‐carbocisteine (72 h) prior to infection reduced the amount of NF‐κB p50 and NF‐κB p65 in nuclear extracts compared to the levels observed in the cells pretreated with vehicle prior to RV14 infection (Table 3). L‐carbocisteine pretreatment also reduced the amount of p50 and p65 that was induced by RV14 infection in the cells obtained from both groups of subjects (Table 3).

## Discussion

In the present study, RV14, a major RV group, was observed to infect HNE cells. Subjects in the allergic group (allergic subjects) displayed bronchial asthma, allergic sinusitis, eosinophilic sinusitis, or atopic dermatitis with a specific serum IgE antibody level greater than 170 IU/mL. Histological examination of the nasal mucosal tissues obtained from allergic subjects showed that eosinophils had infiltrated into the mucosa and submucosa. Cultured cells that were isolated from nasal mucosae positively stained for cytokeratin but not for vimentin in immunocytochemical examinations, confirming that the cells show epithelial cell characteristics. RV14 titers and cytokine concentrations in the supernatants, RV14 RNA replication and susceptibility to RV14 infection were greater in the HNE cells obtained from allergic subjects than in those obtained from non‐allergic subjects (subjects in the non‐allergic group). The RV14 titers and RV14 RNA levels in the cells obtained from allergic non‐asthmatic subjects were also higher than the levels in the cells from non‐allergic subjects. The HNE cells obtained from allergic subjects expressed higher levels of ICAM‐1, the receptor for RV14 11, higher levels of intensity in fluorescently labeled acidic endosomes from which RV14 RNA enters the cytoplasm 14, and higher levels of NF‐κB subunit proteins than cells obtained from non‐allergic subjects. These findings suggest that RV14 replication, susceptibility to RV14 infection and the production of inflammatory cytokines may be stimulated in the HNE cells obtained from allergic subjects partly by the observed increase in ICAM‐1 expression and the increased number of acidic endosomes.

The upregulation of ICAM‐1 expression in airway epithelial cells has been previously reported in asthma patients and monkeys that were stimulated by antigen inhalation 12, 13, 37 and have been associated with airway hyperresponsiveness 12. IL‐4 and TNF‐α, which are released by Th2 lymphocytes, induce ICAM‐1 expression in the airways of non‐atopic and non‐asthmatic subjects 38. Subauste et al. also reported that TNF‐α increased the susceptibility of human airway cells to RV14 infection 2, and we previously reported that IL‐1β ‐induced ICAM‐1 expression in human tracheal epithelial cells 4. In contrast, in the present study, we detected only a small amount of IL‐1β and TNF‐α in the supernatants of HNE cells. Therefore, the amount of IL‐1β and TNF‐α that is released from HNE cells might not be able to induce the upregulation of ICAM‐1 in the HNE cells obtained from allergic subjects. In contrast, before the surgical excision, the nasal tissues from allergic subjects might have been stimulated by IL‐4 and TNF‐α, which is released from cells other than epithelial cells, such as Th2 lymphocytes, as has been observed in atopic asthmatic subjects 39, although we did not examine the function of Th2 lymphocytes using nasal lavage samples. Eosinophils were observed in nasal tissue specimens and these cells might also release cytokines, such as IL‐4 and TNF‐α 40, to stimulate ICAM‐1 expression.

Because ICAM‐1 is the receptor for the major RV types 11, it has been suggested that increased ICAM‐1 expression increases susceptibility to infection with RV in the airway epithelial cells of asthmatic subjects 13. However, the relationship has not been studied. This is the first report to demonstrate that enhancing ICAM‐1 expression increases susceptibility to RV14 infection in HNE cells obtained from allergic subjects. Increased ICAM‐1 expression may also be to blame for the increased frequencies of colds observed in asthmatic adults and asthmatic children in previous reports 41, 42. We also demonstrated that RV14 replication and an associated increase in cytokine production were enhanced in the HNE cells obtained from allergic subjects through an increase in ICAM‐1 expression and a decrease in endosomal pH that was caused by the activation of NF‐κB. The enhanced RV replication and increased cytokine production in the cells of allergic subjects may be components of the mechanisms that contribute to RV infection‐induced asthma exacerbation 1.

Similarly, increased NF‐κB activation was observed in the HNE cells obtained from allergic subjects in the present study. NF‐κB activation has been observed in peripheral blood mononuclear cells in patients with uncontrolled asthma 43. As was previously observed in allergic subjects 44, antigen stimulation may augment NF‐κB activation. However, we did not examine antigen levels in detail in the allergic subjects in this study. Furthermore, similar to the downstream effects of induction of ICAM‐1 expression, increases in the production of IL‐1β, IL‐4, and TNF‐α, which are released from Th2 lymphocytes 38, 39, eosinophils 40, bronchial epithelial cells, and monocytes 45, may be associated with the activation of NF‐κB 46 either before the specimens were surgically excised or while the isolated cells were being cultured. Further studies are required to clarify these mechanisms.

NF‐κB increases the expression of ICAM‐1 and various pro‐inflammatory cytokines 3, 47. We demonstrated that higher protein levels of NF‐κB subunits were observed in the HNE cells obtained from the allergic subjects than in those obtained from the non‐allergic subjects both before and after RV14 infection. Furthermore, both the baseline and infection‐induced levels of IL‐6 and IL‐8 secretion were higher in the HNE cells obtained from the allergic subjects than in those obtained from the non‐allergic subjects. The concentration of sICAM‐1 in the supernatants, the level of ICAM‐1 mRNA expression before infection, the RV14 viral titers, and RV14 RNA replication were higher in the cells obtained from the allergic subjects than in those obtained from non‐allergic subjects. These findings suggest that NF‐κB activation might contribute to enhanced RV14 replication by increasing ICAM‐1 expression in the present study, as previously reported by Schneider et al., who demonstrated that increased RV replication was related to increased ICAM‐1 expression in tracheobronchial epithelial cells that were isolated from COPD patients 48. Increased viral replication was associated with enhanced IL‐6 and IL‐8 production during RV14 infection in the present study, and this effect may be also associated with the development of asthma exacerbations during RV infection 1.

The entry of the RNA of a major RV group, RV14, into the cytoplasm of infected cells is thought to be mediated by destabilizing effects that are induced by receptor binding and endosomal acidification 14. Vacuolar H^+^‐ATPase and Na^+^/H^+^ exchangers regulate endosomal pH 49. In the present study, the specific vacuolar H^+^‐ATPase inhibitor bafilomycin A_1_ reduced RV14 titers and RV14 RNA levels. We demonstrated that the number and fluorescence intensity of the acidic endosomes were increased in the HNE cells obtained from the allergic subjects. The precise mechanisms that contributed to the increase in the number of acidic endosomes are unknown. However, NF‐κB activation might stimulate viral entry into the cell via acidic endosomes because the activation of NF‐κB has been associated with the gene promoter activity of H^+^‐ATPase 50, which induces the entry of H^+^ ions into acidic endosomes 49. Furthermore, we observed that an inhibitor of NF‐κB, CAPE 34, reduced the fluorescence intensity of acidic endosomes and reduced RV14 titers and RV14 RNA levels. Enhanced endosomal acidification might be another mechanism that stimulates RV replication in the cells of allergic subjects.

Increased RV replication has been associated with the reduced induction of type I and type III IFNs in the bronchial epithelial cells of asthmatic patients 8. In contrast, no significant amount of IFN‐γ and IFN‐λ was detected in the present study, similar to our previously reported results in human tracheal epithelial cells 4. Differences between cell types and the composition of culture media might, therefore, result in different levels of production of IFNs.

L‐carbocisteine reduced RV14 titers, cytokine concentrations, and the levels of RV14 RNA in the HNE cells, as was previously shown in human tracheal epithelial cells 15. L‐carbocisteine reduced ICAM‐1 expression, the fluorescence intensity of acidic endosomes, and the activation of NF‐κB. Furthermore, an inhibitor of NF‐κB, CAPE 34, and the vacuolar H^+^‐ATPase inhibitor bafilomycin A_1_ 35 also reduced RV14 titers and RV14 RNA replication, as was previously reported in human tracheal epithelial cells 36. Therefore, L‐carbocisteine may inhibit RV14 infection in HNE cells partly by reducing ICAM‐1 levels and the number of acidic endosomes by inhibiting the activation of NF‐κB, as was previously reported in experiments using human tracheal epithelial cells 15. The anti‐inflammatory effects of L‐carbocisteine may, therefore, be associated with the ability of this agent to prevent the exacerbation of bronchial asthma 22, 23.

RV infection has been associated with the acute exacerbation of bronchial asthma 1, and carbocisteine reduces the frequency of asthma exacerbations 22, 23. Therefore, in the present study, we examined the effects of L‐carbocisteine on RV14 replication and the release of cytokines after RV14 infection in HNE cells to study the mechanisms involved in the clinical effects of this agent that are associated with preventing RV infection‐induced asthma exacerbation. Further studies (in either animal models or human trials) are required to confirm the potential value of using L‐carbocisteine in RV infections and to prevent RV infection‐induced asthma exacerbation.

We previously published a study that described the effects of L‐carbocisteine on endosomal acidification, ICAM‐1 expression, RV replication, and RV‐induced cytokine production in primary cultures of human tracheal epithelial cells 15. Although the mucolytic agent L‐carbocisteine has been shown to provide clinical benefits in patients with bronchial asthma, including improvements in symptoms and quality of life 17 and a reduction of exacerbation 22, 23, it remained unclear whether L‐carbocisteine inhibited RV replication or RV‐induced cytokine production in airway epithelial cells in allergic subjects. In the current study, we demonstrate that L‐carbocisteine reduces RV replication and the RV infection‐induced production of cytokines in HNE cells obtained from allergic subjects.

There are some limitations to the present study. In the present study, the HNE cells were cultured under immersed feeding conditions, as reported by Wark et al. 8. However, Jakiela et al. 51 demonstrated that Th2 cytokines reduced RV replication in human bronchial epithelial cells that were cultured on filter membranes using an air‐liquid interface method. In contrast to what has been observed in proliferating epithelial cells that are maintained in a typical submerged adherent cell culture, cells cultured using an air–liquid interface method on filter membranes develop a differentiated pseudo‐cylindrical epithelial barrier in which ciliated and mucosal cells can mature 51. ICAM‐1 expression could also depend on differentiation. Therefore, we cannot rule out the possibility that the differences in the proliferation and maturation of the cultured cells may have influenced differences in RV14 replication or susceptibility to RV14 infection between allergic and non‐allergic epithelia. The immersed feeding culture condition is, therefore, the first limitation of this study.

The specific serum IgE antibody levels were greater than 170 IU/mL in all of the subjects in allergic group (allergic subjects) against at least one common inhalant allergen, and significant eosinophil infiltration was observed in the mucosae and submucosae in the specimens obtained from the allergic subjects. Furthermore, we confirmed that all of the subjects in the non‐allergic control group (non‐allergic subjects) had low specific serum IgE antibody levels, and no significant eosinophil infiltration was observed in the mucosal specimens obtained from non‐allergic subjects. However, in this study, the allergic group included subjects with bronchial asthma, allergic sinusitis, eosinophilic chronic sinusitis, and atopic dermatitis. The non‐allergic group included subjects with sinus cyst, sinus mycosis, papilloma, chronic sinusitis, cerebrospinal fluid rhinorrhea, nasopharyngeal cancer, and a sphenoid bone tumor. Thus, the characteristics of the subjects in the allergic and non‐allergic groups were heterogeneous, and the variation in the features and the different disease statuses of the subjects in both groups represent the second limitation of this study.

We demonstrate that L‐carbocisteine reduced RV14 replication in HNE cells. However, a number of other drugs, including inhaled steroids, β_2_ agonists and leukotriene receptor antagonists, also reduce asthma exacerbations 52. Furthermore, inhaled steroids and β_2_ agonists inhibit RV replication and the RV infection‐induced production of cytokines, as we previously reported 26, 31, 53. Therefore, careful attention is needed when considering the influence of the effects of these drugs in evaluations of the ability of L‐carbocisteine to prevent asthma exacerbation.

Studies in vivo have not shown any differences in viral load between asthmatics and controls 54. Furthermore, higher expression of ICAM‐1 in vivo could also mean higher levels of soluble ICAM‐1 55. These findings suggest that higher ICAM‐1 expression may lead to the neutralization of RV and improved viral loads. Therefore, the findings that we observed in an ex vivo study using the cultured HNE cells may not explain in vivo findings. This is the fourth limitation of this study.

In conclusion, evaluations of the results of this study are limited because the baseline diseases of the subjects from whom the cells were obtained were heterogeneous and because we used a cellular model that consisted of culturing the cells under immersed feeding conditions. However, increased RV14 replication was observed in the nasal epithelial cells obtained from allergic subjects, which might be partly attributed to the association of RV14 infection with increased ICAM‐1 expression and the increased number of acidic endosomes. L‐carbocisteine may modulate rhinovirus replication and infection‐induced airway inflammation in allergic subjects.

## Conflict of Interest

All authors have no conflicts of interest. Yamaya is a Professor, Kubo is an Associate Professor and Ms. Lusamba Kalonji is a laboratory assistant in the Department of Advanced Preventive Medicine for Infectious Disease, Tohoku University Graduate School of Medicine. This department had been funded by 11 pharmaceutical companies until 31 March 2014, including Kyorin Pharmaceutical Co., Ltd. which provided L‐carbocisteine. From 1 April 2014, this department is funded by eight pharmaceutical companies, which are as follows: Kyorin Pharmaceutical Co. Ltd., Abott Japan, Co., Ltd., Taisho Toyama Pharmaceutical Co., Ltd., AstraZeneca Co. Ltd, Otsuka Pharmaceutical Co. Ltd., Teijin Pharma Co., Ltd., Toyama Chemical Co., Ltd., and Nippon Boehringer‐Ingelheim Co., Ltd.
